# Special Issue “Functional Nanomaterials: Structures, Compositions and Various Applications”

**DOI:** 10.3390/ijms27062819

**Published:** 2026-03-20

**Authors:** Magdalena Laskowska

**Affiliations:** Institute of Nuclear Physics Polish Academy of Sciences, PL-31342 Krakow, Poland; magdalena.laskowska@ifj.edu.pl

Research into functional nanomaterials lies at the heart of many technological breakthroughs because it connects fundamental structure–property relationships with real-world performance in devices and processes. In particular, nanomaterials combine the advantages of nanoscale dimensions with the versatility of surface functionalization using tailored molecular or ionic motifs. At the nanoscale, the high surface-to-volume ratio and size-dependent electronic structure can enhance reactivity, charge transport, and optical response, enabling strong catalytic, sensing, and light–matter interaction effects [1,2]. Surface functionalization further expands this toolbox by allowing for precise control over surface chemistry, elemental composition at the interface, wettability, and specific binding interactions, while also improving dispersion, stability, and compatibility with polymers or biological environments. Together, these features make functional nanomaterials powerful design platforms for applications ranging from energy conversion and storage to advanced coatings, diagnostics, and environmental remediation [3].

Against this backdrop, the Special Issue “Functional Nanomaterials: Structures, Compositions and Various Applications” brings together cutting-edge research and forward-looking perspectives on nanoscale materials engineering and the physico-chemical principles that enable controllable functionality. This topic is particularly timely because many of today’s most pressing challenges, such as clean energy generation and storage, low-power electronics, efficient catalysis and chemical manufacturing, environmental remediation, and advanced biomedical technologies, are increasingly limited by materials performance rather than by conceptual design alone. Nanomaterials offer an exceptional degree of tunability: small, deliberate changes in size, morphology, crystal structure, defect density, composition, and surface chemistry can translate into large shifts in electronic structure, transport behavior, reactivity, and interfacial phenomena. At the same time, this sensitivity underscores the need for mechanistic understanding, as reproducibility, long-term stability, scalability, and safety remain key barriers to real-world implementation. Bridging fundamental insight with rational design principles is therefore essential to move beyond isolated demonstrations toward robust, predictable, and application-ready materials. By highlighting advances that connect structure and composition to measurable function across diverse use cases, this Special Issue aims to define current best practices and outline promising directions for the next generation of functional nanomaterials.

In this Special Issue, readers will find a broad yet cohesive snapshot of how functional nanomaterials are designed, understood, and deployed across diverse application domains, illustrating how nanoscale and molecular-level materials engineering translates into new opportunities in electronics, energy technologies, environmental remediation, and bio-inspired solutions. The contributions span a broad palette of material classes and functions: from low-dimensional carbon-based platforms (graphene, carbon nanotubes, and nanoporous carbons) and their hybrids with functional molecules (single-molecule magnets), through porphyrin-based metal–organic frameworks (P-MOFs) for visible-light-enabled water remediation, to polymer composites reinforced with boron nitride and boron nitride nanosheets for thermal management, as well as engineered oxide matrices obtained by metal anodization (e.g., Al, Ti, Nb, Zn, or Ta) in the form of highly ordered nanoporous layers and nanotube arrays. Collectively, the papers address (i) surface and molecular engineering strategies for creating advanced functional interfaces (e.g., modified anodized metal matrices and electrochemical graphene–water interfaces), (ii) environmentally focused studies on contaminant capture and transport (including nanoplastics behavior in porous media, data-driven prediction of antibiotic adsorption on nanoporous carbon, and photocatalytic/adsorptive pollutant removal using MOFs), and (iii) structure-driven performance in next-generation materials (such as bio-inspired thermally conductive fibers and nanostructured platforms for the controlled deposition of single-molecule magnets (SMMs)). Complementing these experimental advances, several contributions emphasize mechanistic and data-driven perspectives, showing how modern computational and machine-learning tools (including QnSPR models) can guide rational optimization of pore texture, interfacial chemistry, and functional performance across energy, environmental, and advanced-device contexts.

The Special Issue opens with three review articles that set the scene for how controlled structure and interfaces can be used to program functionality at the nanoscale. The first review, by M. Adamek et al., titled “Nanostructures as the Substrate for Single-Molecule Magnet Deposition”, offers a comprehensive overview of strategies for anchoring single-molecule magnets (SMMs) onto various nanostructured surfaces, an essential step in the development of molecular spintronics and next-generation information technologies (Contribution 1). The authors detail the quantum-mechanical origins of magnetic behavior in representative SMM classes, such as Mn_12_, Fe_4_, chromium rings, and lanthanide phthalocyanines, highlighting unique features like magnetic hysteresis and slow relaxation. The review categorizes potential substrates by dimensionality (0D to 3D), encompassing nanoparticles [4,5], carbon nanotubes, graphene [6], and porous matrices like metal–organic frameworks (MOFs) [7] and mesoporous silica [8], while analyzing how surface–molecule interactions influence magnetic anisotropy and quantum tunneling [9]. A primary challenge addressed is the refinement of deposition methods—including chemisorption, physisorption, and encapsulation—to achieve precise molecular organization while preserving the initial functionality and properties of the magnets for applications in quantum computing and high-density memory storage.

Complementing this molecular-level perspective, the second review surveys engineered metal oxide matrices produced by anodization of metals such as aluminum, titanium, niobium, zinc, and tantalum. M. Schabikowski et al., in their publication “Beyond Oxidation: Engineering Functional Anodised Metal Matrices Through Molecular and Surface Modifications”, provide a comprehensive overview of how electrochemical anodization can transform various metals into highly structured, multifunctional oxide platforms (Contribution 2). The authors detail the synthesis and properties of diverse architectures, including ordered nanoporous arrays, nanotubes, and nanowires, which are derived from a wide range of substrates such as aluminum, titanium, niobium, zinc, tantalum, magnesium, and iron. A central focus of the review is the use of post-anodization modification strategies, such as chemical functionalization [10], metal/non-metal doping [11], and thin-film deposition [12] to expand the utility of these materials far beyond simple corrosion protection [13]. By leveraging the synergy between structural precision and molecular-level surface engineering, these matrices are being developed for advanced applications in energy storage (e.g., lithium-ion batteries), environmental remediation and biomedical engineering.

Several subsequent contributions demonstrate how rational nanostructure design translates into functional performance across different application spaces. In the context of environmental technologies, one review addresses visible-light-driven water remediation using porphyrin-based metal–organic frameworks (P-MOFs). In the review “Recent Developments in Porphyrin-Based Metal–Organic Framework Materials for Water Remediation under Visible-Light Irradiation”, N. K. Shee et al. provide a comprehensive evaluation of porphyrin-based metal–organic frameworks (P-MOFs) as multifunctional scaffolds for the adsorptive and photocatalytic removal of pollutants (Contribution 3). The authors outline major fabrication routes for P-MOFs (with solvothermal synthesis as the dominant method) and show how porphyrin linkers enable tuning of pore structure, topology, and optoelectronic properties via peripheral modification and metal-node selection. The review highlights structure–activity relationships behind visible-light photodegradation, where improved light harvesting and charge separation promote reactive oxygen species generation and pollutant mineralization [14,15]. It also identifies key bottlenecks such as carrier recombination, limited water/chemical stability, and cost/scale-up of porphyrinic ligands, and points to composite P-MOFs as a main strategy to boost stability and photocatalytic efficiency for practical wastewater treatment [16,17].

A different route to environmental protection is explored through adsorption and retention processes, where two papers focus on pollutant capture and transport in porous media. The publication “Nanomaterial Texture-Based Machine Learning of Ciprofloxacin Adsorption on Nanoporous Carbon” explores the use of data-driven machine learning to predict the removal of the antibiotic ciprofloxacin from aqueous solutions using specialized carbon adsorbents (Contribution 4). In this study, M. Käärik et al. analyzed a library of 87 carbide-derived carbons (CDCs) synthesized from diverse precursors and observed that ciprofloxacin adsorption capacities span a broad range (55–495 mg g^−1^), reflecting differences in porous architecture. A key outcome is a multi-linear Quantitative nano-Structure–Property Relationship (QnSPR) model that captures the adsorption trends using three textural descriptors: the specific surface area (S_dft_) and the pore-volume contributions associated with pores in the 1.1–1.2 nm and 3.3–3.4 nm size ranges. The model reveals a physically meaningful correlation: the 1.1–1.2 nm pores are in good agreement with the smallest molecular dimension of ciprofloxacin (~1.00 nm) and show a strong positive association with uptake, whereas the 3.3–3.4 nm pores help account for materials with low volume in the smaller-pore window and could accommodate ciprofloxacin as dimers or trimers. Using quenched solid density functional theory (QSDFT) to quantify pore textures, the study illustrates how nanoporous carbons can be rationally tuned for removing pharmaceutical residues from water [18,19].

Complementarily, another work examines how magnetic biochar and solution chemistry control the retention and release of nanoplastics in porous media. In the publication “Coupled Influence of Magnetic Biochar and Solution Chemistries on Retention and Release of Nanoplastics in Porous Media”, Y. Qin et al. investigate the environmental fate of functionalized nanoplastics (NPs), specifically those with carboxyl (-COOH) and amino (-NH_2_) groups, within saturated porous media such as quartz sand (Contribution 5). The research highlights that the addition of magnetic biochar (MBC) significantly inhibits NP mobility by increasing collector surface roughness and providing additional chemical adsorption sites [20]. Furthermore, the study explores the synergistic effects of solution chemistry, demonstrating that humic acid (HA) promotes NP transport through electrostatic and steric effects, while divalent cations (Ca^2+^) enhance retention via cation bridging and charge shielding [21,22]. By analyzing NP release under varying ionic strength and pH levels, the authors differentiate between reversible retention in shallow minima and irreversible trapping in deep primary minima, providing critical insights for the remediation of soil and groundwater contaminated by emerging plastic pollutants [23].

Building on the importance of nanoscale interfaces highlighted in the reviews, one of the research papers provides fundamental insight into charge dynamics at electrochemical boundaries, focusing on the graphene–water interface. The publication by A. V. Butko et al., “Dirac Electrons with Molecular Relaxation Time at Electrochemical Interface between Graphene and Water”, explores the time dynamics of charge accumulation at the graphene–water interface, a factor critical for the efficiency of energy storage and sensing devices (Contribution 6). Utilizing impedance spectroscopy across a wide frequency range (0.02 Hz to 50 kHz), the authors show that the measured capacitance *C*_*m*_ exhibits two distinct regimes: at high frequencies (1–50 kHz) it is limited by the rapid transport/response of molecular charges in water reaching the graphene electrode, whereas below ~300 Hz the dominant contributions come from the electric double-layer capacitance and the graphene quantum capacitance, with the observed dispersion explained by molecular relaxation of these capacitances. From the voltage dependence they further infer that Dirac-cone reconstruction requires a relaxation time longer than 0.1 s, pointing to slower interfacial processes likely involving quasi-chemical bonding between Dirac electrons in graphene and molecular charges at the interface; as a result, these Dirac electrons behave as quasiparticles with an effective mass on the order of the mass of water molecules. The findings are relevant for understanding interfacial charging/discharging and for potential applications in supercapacitors and selective chemical and biological sensors [24,25].

Finally, the Special Issue also addresses a key materials bottleneck in modern electronics: thermal management. In the work “Bio-Inspired Thermal Conductive Fibers by Boron Nitride Nanosheet/Boron Nitride Hybrid”, J. Zhang et al. detail the development of advanced composite fibers designed to address heat dissipation challenges in modern electronic devices (Contribution 7). The researchers utilized a polyurethane (PU) matrix reinforced with a hybrid of boron nitride (BN) and surface-grafted boron nitride nanosheets (BNNSs). By employing a combination of wet-spinning and hot-pressing methods, they achieved a unique bio-inspired architecture where the BN fillers are horizontally aligned along the fiber axis, creating efficient, continuous pathways for phonon transport. This engineered internal–external interconnected structure resulted in a significant 176.47% thermal conductivity enhancement (TCE) compared to pure polymer fibers, while simultaneously maintaining excellent mechanical flexibility and chemical resistance. The study demonstrates that these hybrid fibers provide a highly effective strategy for the fabrication of next-generation thermal interface materials (TIMs), essential for preventing overheating in high-performance electronic systems [26,27].

Taken together, the seven contributions in this Special Issue demonstrate that progress in functional nanomaterials increasingly depends on linking nanoscale structure and composition to measurable functionality through a mechanistic, interface-aware design approach. Across molecular spin systems and engineered oxide matrices, the papers highlight the importance of controlled immobilization, ordered architectures, and surface modification as enabling strategies for device-relevant performance (Contributions 1 and 2). At the same time, the collection underscores that interfacial phenomena can dominate macroscopic behavior, as shown by unconventional charge dynamics at the graphene–water boundary with implications for electrochemical technologies (Contribution 6). The issue also illustrates how functionality can be tailored toward pressing societal needs, ranging from visible-light photocatalysis for pollutant degradation to adsorption-driven removal of emerging contaminants and mitigation of nanoplastics transport, where performance is governed by a combination of nanostructure, chemistry, and environmental conditions (Contributions 3, 4 and 5). Finally, the included work on thermally conductive polymer composites emphasizes that translating nanoscale design into robust, scalable materials remains essential for thermal management in modern electronics (Contribution 7). Overall, these studies collectively point toward a unifying theme: predictable and application-ready functionality emerges when materials are engineered together with their interfaces, operating environments, and structure–property relationships in mind.

Looking ahead, several research directions appear particularly promising for advancing functional nanomaterials toward reliable and scalable applications. First, greater emphasis on standardized characterization and operando and in situ methods will be critical for connecting nanoscale descriptors (defects, surface states, pore architectures, interfacial bonding) to performance under realistic working conditions, especially for electrochemical interfaces and catalytic environments (Contributions 3 and 6). Second, the field would benefit from more systematic strategies for interface engineering, including controlled immobilization of molecular functionalities on low-dimensional supports and stable surface modification routes for ordered oxide platforms, with a particular focus on preserving function over time and under device-relevant stimuli (Contributions 1 and 2). Third, bridging fundamental discovery with deployment requires tackling reproducibility, long-term stability, and scalability: photocatalysts must retain activity across cycles and complex water matrices, adsorption materials must maintain capacity and selectivity in multicomponent systems, and polymer-based thermal interface materials must combine high conductivity with processability and mechanical robustness (Contributions 3, 4, and 7). Fourth, data-driven approaches should increasingly evolve from prediction to closed-loop design, where machine learning models are paired with targeted synthesis and validation to accelerate optimization, particularly for porous carbons and other compositionally complex systems where intuition alone is insufficient (Contribution 4). Fifth, environmental and safety considerations deserve continued attention, including mechanistic understanding of nanoplastics behavior in porous media and the design of functional materials that are not only effective, but also minimize unintended persistence or secondary contamination (Contribution 5). Finally, the collection suggests that cross-fertilization between subfields such as quantum/molecular functionalities, electrochemical interfaces, photocatalysis, porous adsorbents, and thermal-management composites can yield transferable design rules for tuning transport, reactivity, and stability through controlled nanostructure and composition (Contributions 1, 6, and 7). By advancing mechanistic insight, standardized evaluation, and scalable fabrication in parallel, future work can help translate the rich tunability of nanomaterials into robust technologies aligned with real-world constraints.
